# Expert consensus on the use of oropharyngeal probiotic Bactoblis in respiratory tract infection and otitis media: available clinical evidence and recommendations for future research

**DOI:** 10.3389/fped.2024.1509902

**Published:** 2025-01-28

**Authors:** Qiang Wang, Yatong Zhang, Xiaoling Cheng, Zhi Guo, Yang Liu, Li-hong Xia, Zhigang Liu, Junqing Zheng, Zihe Zhang, Kai Sun, Guanxin Shen

**Affiliations:** ^1^ Department of Immunology of College of Medicine, Wuhan Wuchang Hospital, Wuhan University of Science and Technology, Wuhan, China; ^2^ Department of Pharmacy, Beijing Hospital, Beijing, China; ^3^ Department of Pharmacy, Beijing Children’s Hospital, Capital Medical University, Beijing, China; ^4^ Huazhong University of Science and Technology Union Shenzhen Hospital, Shenzhen, China; ^5^ Pediatric Department, Wuhan Asian General Hospital, Wuhan, China; ^6^ Pediatric Department, Affiliated Hospital of Shandong University of Traditional Chinese Medicine, Jinan, China; ^7^ Pediatric Department, Jinan Maternity and Child Care Hospital, Jinan, China; ^8^ Department of Otolaryngology Head and Neck Surgery, Shandong Maternity and Child Care Hospital, Jinan, China; ^9^ Department of Obstetrics and Gynecology, First Affiliated Hospital of Shandong First Medical University, Jinan, China; ^10^ Department of Immunology, Tongji Medical College Huazhong University of Science and Technology, Wuhan, China

**Keywords:** recurrent respiratory tract infections, rational use of antibiotics, otitis media, streptococcal pharyngitis/tonsilitis, oropharyngeal probiotic Bactoblis

## 1 Introduction

The upper respiratory tract is a crucial site for host defense, as it is home to bacterial communities that both modulate host immune defense and serve as a reservoir of potential pathogens. During the first few years of life, much like the gastrointestinal tract microbiome, nasopharyngeal microbiota in young children changes from an immature state to a more diverse state as it matures to resemble the adult microbiota, resulting in a higher risk of respiratory illness in young children (1). According to a systematic analysis of respiratory infections in 33 provinces in China from 1990 to 2019, a consistently increasing trend in the number of RTi cases was observed. RTi cases in 2019 were 3%, 5%, and 11% higher than those in 2010, 2000, and 1990, respectively. The incidence rate of URTis was the largest in younger children indicating that the future URTi and LRTi prevention strategies should focus on maternal and child health, especially in young children (2). URTIs, although they are generally mild and resolve spontaneously, can significantly impact the quality of life, school attendance of children, and work absence for caregivers. Pediatricians and caregivers require prevention strategies to reduce the recurrence incidence in children prone to frequent RTIs to reduce medical visits. Risk factors that are significantly associated through odds ratio (OR) with recurrent respiratory tract infection include asthma [OR = 8.31 (*P* < 0.001)], allergy [OR = 2.31 (*P* < 0.001)], initial use of antibiotics [OR = 1.72 (*P* < 0.001)], breastfeeding duration <6 months [OR = 1.24 (*P* < 0.002)], and maternal body mass index 1.19 (*P* < 0.001) (3). In general, distinct microbial maturation patterns involved with early asymptomatic respiratory viral presence and dynamics in gene expression profiles (4) translate into either a beneficial microbiota or susceptibility to RTI development and/or asthma and/or allergic rhinitis. A “three-hit” model for chronic RTI development begins with an establishment of non-beneficial respiratory microbiota as the first hit, suffering RTi with varying degrees of severity as the second hit, and progression to a long-term inflammatory airway status as the third hit (5). The strategies for managing pediatric recurrent respiratory tract infections (RRTi) can be built on several key approaches. One approach focuses on modulating non-specific immune responses to strengthen the body's natural defenses against infectious agents. Another involves enhancing specific immune responses to directly combat respiratory pathogens. Additionally, managing inflammation through anti-inflammatory responses can help reduce infection- or pathogen-induced airway inflammation. Modulating respiratory microflora through the administration of probiotics that colonize the airway is also promising. This approach aims to improve the interaction between host commensal bacteria and the immune system, ultimately reducing susceptibility to RRTi.

To date, unnecessary antibiotics are widely prescribed for pediatric RTIs and most pediatric patients with RTIs do not receive guideline-recommended antibiotic classes in Chinese primary healthcare facilities (6). Meanwhile, parents often resort to self-medication with antibiotics for their children in China (7). Unfortunately, microbiome recovery lost diversity after short courses of antibiotics and can be delayed by azithromycin; the long-term effect of altered diversity, resistance, and composition is considered “antibiotic scarring” (8).

Well-documented evidence suggests that early antibiotic exposure affects the development of infant gut microbiota and the disturbances in the host increase the susceptibility to a variety of diseases later in life including respiratory infections (9). In addition, antibiotic use in early life preferentially impairs the development of lung mucosal-associated invariant T (MAIT) cells which plays an important role in recognizing a broad array of respiratory pathogens (10).

Antibiotic-induced microbial disruption in early life can be exacerbated by the vertical transmission of resistance genes from mother to offspring during pregnancy and lactation (11) and is associated with higher risks of childhood metabolic disorders (12); neurobehavioral conditions including autism spectrum disorder, intellectual disorder, language disorder, and epilepsy (13); and juvenile idiopathic arthritis (JIA) (14).

Although most recent reviews reveal variable outcomes, knowledge gaps, and insufficient evidence to recommend probiotics for the prevention or management of RTi conditions (15–18), a specific formula with consistent positive clinical results should be recommended and be considered a possible supportive approach in selected patients to improve their quality of life and reduce the burden of RTIs.

Oropharyngeal probiotic Bactoblis has clinical and real-world evidence for indications of selected respiratory disorders in children, adolescents, and adults. These clinical studies were technically reviewed to provide insight into the potential of oropharyngeal probiotics as a clinical modality targeted at recurrent and non-recurrent respiratory infectious diseases. As published data demonstrate that only a single formulation of oral probiotic species complies with the required stability requirements established by Chinese medical practitioners, other evidence was excluded from the examined information used as a basis for this consensus. Indeed, investigators of a New Zealand study of a *Streptococcus salivarius* K12 strain for prevention of the development of symptomatic Group A *Streptococcus pharyngitis* faced confounding factors arising from their choice of the final product used in the study, including contamination of the placebo formulation with active strain administered to the test group (19). These observations underscore the importance of selecting clinically validated probiotic formulas that meet established standards, to ensure efficacy and reliability in managing respiratory health.

Therefore, it is extremely important for healthcare professionals to be properly educated and updated on the knowledge of oropharyngeal probiotics even though their awareness toward gut probiotic has been well-established (20), with the purpose of assisting pediatricians or practitioners who are willing to offer their patients an alternative option to safely and effectively manage their respiratory conditions and appropriately recommend the use of oropharyngeal probiotics. The following professional recommendations are meant to be broadly applicable and should be viewed as the preferred alternative and adjunctive approach. However, they are not meant to replace standard approaches and clinical management strategies depend on individual clinical scenarios. This project aims to support decision-making in respiratory health based on scientific evidence and the daily life of both the pediatric patients with respiratory infections and their caregivers.

A meeting of our consensus panel consisting of clinical and scientific experts (pediatrician, obstetrician and gynecologist, oncologist, pharmacist, immunology and microbiology scientists) was convened to review, examine, select, and synthesize 38 evidence-based publications on oropharyngeal probiotics, which were organized into two main topics, which are the existing evidence-based science and the recommendation for future researches. Participants in the meeting jointly considered key questions and generated and approved the outcomes hereby summarized. We hope that this consensus statement will provide consensus views on the appropriate use of oropharyngeal probiotics and recommendations for future research associated with oropharyngeal probiotics and human respiratory health.

## 2 Expert consensus statement based on existing evidence-based science

### 2.1 Recurrent respiratory tract infections

Very few convincing dietary measures for reducing the frequency and clinical relevance of recurrent respiratory episodes in RTi-prone children have been developed. Until July 2024, professional societies and groups of experts technically reviewed 10 clinical trials conducted with oropharyngeal probiotics with documented efficacy for the management of RRTi published since 2012 with a total of 832 children participating. Oropharyngeal probiotic Bactoblis was given to subjects in the form of slowly dissolving oral lozenges, to be administered before bedtime after brushing their teeth every evening, and they were required to suck the lozenge until fully dissolved (approximately 4–5 min) and not to chew or directly swallow it. They were suggested not to drink or swallow any substance for at least 1 h after the administration of oropharyngeal probiotic lozenges. If the subjects were prescribed with antibiotics by study practitioners during RTIs, they are requested to continuously take the oropharyngeal probiotics during the days taking antibiotics, but making sure that oropharyngeal probiotics and antibiotics had been taken 2 h apart. Oropharyngeal probiotics with recommendations for use in clinical practices were discussed based on the evaluation of 10 trials in terms of population, burden of RRTi, current treatment strategies, probiotic dose, and administration schedule. Overall, oropharyngeal probiotic, as a general group, reduced the risk of developing new episodes of respiratory tract infections and antibiotic exposure in children with RRTi. A conditional recommendation based on level 2 evidence criteria graded by study type of randomized trials with consistent effect according to the latest World Gastroenterology Organisation Global Guidelines: Probiotics and Prebiotics 2024 (21), on the adjunctive use of oropharyngeal probiotics supportive of standard treatment in children with recurrent respiratory tract infections was made, in the context of the corresponding 10 clinical trials. Most of the 10 trials are homogeneous in regards to study subjects, dosage, schedule of administration, and formulation. Oropharyngeal probiotic Bactoblis has been found to have a good safety profile. These findings revealed that compared to controls, people with a history of recurrent RTIs receiving oropharyngeal probiotic Bactoblis had a significantly lower risk of new episodes of RTi during the study period reduced by around 90%. The protective benefit was observed during both the intervention period and maintained several months after the end of the intervention. The risk of antibiotic exposure was significantly higher among controls than among subjects administrating oropharyngeal probiotic Bactoblis. The limitations of the 10 studies were the use of non-blinded method and that the pathogen type and severity of RTIs were not defined by molecular tests and RTi-related quality of life. The type of antibiotic used by the subjects were not described in the article, and the size of the studies was generally small. However, the baseline clinical characteristics of the enrolled subjects were precisely defined in all of 10 trials. Among the 10 trials, 6 were published in English, of which 5 were carried out in Europe and 1 was carried out in China, and 4 were carried out in Ukraine and published in Ukrainian with the abstract in English and in most cases were published in Ukrainian journals. Clinical studies evidencing that oropharyngeal probiotic Bactoblis can provide clinical benefit on recurrent RTi management are listed in Table 1.

**Table 1:** Studies providing evidence that the oropharyngeal probiotic formula, Bactoblis, can provide clinical benefit on recurrent RTi management.

Reference	Year	Subjects	Age	Clinical evidence^a^
Di Pierro et al (22).	2012^b^	82	3–12	Among 65 children with a history of recurrent streptococcal respiratory infections, 45 were treated with oropharyngeal probiotics for 90 days, and the remaining 20 subjects who did not take any probiotics served as the recurrent control group. Another 17 children without recurrent respiratory infections served as the healthy control group. The incidence of streptococcal pharyngotonsil infection decreased by 92% (*p* < 0.0001) compared to that of the previous year during the 90-day oropharyngeal probiotic administration, while it increased by 39% (*p* < 0.001) in the recurrent control group. In contrast, there was a non-significant increase of 29% in the incidence of streptococcal pharyngotonsil infection among healthy control children compared to that of the previous year. During the 6-month follow-up period, children with recurrent streptococcal respiratory infections who received oropharyngeal probiotics experienced a significant reduction of 66% (*p* = 0.0278) in the incidence of pharyngeal tonsillitis compared to children in the recurrent control group
Di Pierro et al (23).	2013	40	18–65	Twenty adult patients with streptococcal recurrent pharyngitis or tonsillitis were treated with oropharyngeal probiotics for 90 days as the probiotic group and 20 of whom did not take probiotics as the control group. The incidence of pharyngeal tonsillitis in adults during oropharyngeal probiotic administration decreased by 84% (*p* < 0.001) compared with that of the previous year, while it increased slightly by 14% (*p* < 0.001) in the control group. During the 6-month follow-up period, the incidence of pharyngeal tonsillitis in the probiotic group was 62% lower than that in the control group (*p* = 0.0389)
Di Pierro et al (24).	2014^b^	60	3–13	The prevalence of streptococcal pharyngotonsillitis decreased significantly by 96.8% (*p* < 0.001) during the 90-day oropharyngeal probiotic administration compared with the same quarter of the previous year in children with recurrent streptococcal pharyngotonsillitis, while the prevalence of viral pharyngeal tonsillitis was significantly reduced by 80% (*p* < 0.01). The prevalence of streptococcal pharyngotonsillitis and viral pharyngotonsillitis in the control group was not significantly different from that in the previous year. Furthermore, the days of medication, absence from school for children, and absence from work for parents due to caregiving to their children were reduced during the study
Di Pierro et al (25).	2016^b^	124	3–10	Forty-eight children with recurrent streptococcal adenoiditis were given oropharyngeal probiotics for 90 days, and another 76 children without recurrent adenoiditis were enrolled as healthy controls. The prevalence of adenoiditis in children was significantly reduced by 89.6% (*P* < 0.01) during oropharyngeal probiotic administration compared to that in the previous year, while it increased by 33.3% non-significantly in healthy controls. Prevalence of various respiratory tract infections in children was significantly lower during oropharyngeal probiotic administration compared with that of healthy controls, including a 93% (*P* < 0.01) reduction in bronchitis, 76% (*P* < 0.01) reduction in viral pharyngitis, 69% (*P* < 0.05) reduction in rhinitis, 95% (*P* < 0.01) reduction in influenza, 93% (*P* < 0.01) reduction in laryngitis, and 100% (*P* < 0.01) reduction in acute otitis media
Gregori et al (26).	2016^b^	130	3–7	Seventy-six children with recurrent streptococcal pharyngotonsil were treated with oropharyngeal probiotics for 90 days, and 54 children took no probiotics as the control group and were followed up for 9 months. During the 1-year observation period, administration of oropharyngeal probiotics for 3 months significantly reduced the prevalence of streptococcal pharyngotonsil infection by 82% compared to children in the control group (*p* < 0.001)
Kryuchko et al (27).	2017	66	3–10	Pediatric outpatients were classified as three subgroups according to the diagnosis, including 26 in the recurrent pharyngotonsil infection group, 22 in chronic adenoid or tonsil hypertrophy group, and 18 in beta-hemolytic Group A *Streptococcus* (BHSGA) infection group, and children in each subgroup were further assigned to take oropharyngeal probiotics for 30 days or to take no probiotic as controls and were followed up for 5 months. In the BHSGA subgroup, the prevalence of BHSGA pharyngotonsil infection in children taking oropharyngeal probiotics during the study period was 90% lower than that in the previous year (*p* < 0.001) and was 86% lower than that in control children (*p* < 0.001). In the chronic adenoid or tonsil hypertrophy subgroup, oropharyngeal probiotics in combination with medication significantly improved the severity of chronic adenoiditis-associated symptoms, such as nasal dyspnea, mouth breathing, and snoring during sleep, cough (mainly at night and in the morning), nasal congestion, and hearing loss by about two times, compared with children using only a drug prescription. In the recurrent pharyngotonsil infection subgroup, significant changes in the pharyngeal microbiome composition were observed during oropharyngeal probiotic administration, especially the decrease in the detection rates of pathogenic *Haemophilus influenzae*, *Staphylococcus aureus*, *Streptococcus pneumoniae*, and *Streptococcus pyogenes*, whether been compared to the time before the study started or been compared with the control group, while the risk of various respiratory infections was lower than that in the control group, including a 75% reduction in the incidence of viral pharyngitis (*p* < 0.01), 92% reduction in the incidence of bronchitis (*p* < 0.01), 65% reduction in the incidence of rhinitis (*p* < 0.05), 86% reduction in the incidence of laryngitis (*p* < 0.01), and 100% reduction in the incidence of otitis media (*p* < 0.01). During the entire 6-month study period, it was observed that the protective effect of 30-day oropharyngeal probiotic administration may last for a few months after cessation of oropharyngeal probiotic administration, while this protective benefit decreased during the fourth month
Kryuchko et al (28).	2018^c^	48	3–14	Pediatric outpatients diagnosed with recurrent adenoiditis or recurrent respiratory infections were treated with two adjuvant courses of oropharyngeal probiotics for 30 days in each course with a 90-day interval and were followed-up for 6 months of clinical observation. Compared to the same time of previous year, the incidence of streptococcal adenoiditis in children treated with two adjuvant courses oropharyngeal probiotics decreased by 87% during the study period compared to that in the previous year (*P* < 0.001), the incidence of non-streptococcal caused adenoiditis was reduced by 90% (*P* < 0.001), the incidence of acute otitis media was reduced by 70% (*P* < 0.001), the incidence of viral pharyngitis was reduced by 67% (*P* < 0.01), the incidence of bronchitis was reduced by 94% (*P* < 0.01), the incidence of rhinitis was reduced by 61% (*P* < 0.05), the incidence of influenza was reduced by 81% (*P* < 0.01), the incidence of laryngitis was reduced by 81% (*P* < 0.01), and the incidence of stomatitis was reduced by 83% (*P* < 0.01). In addition, the number of days taking antibiotics decreased by 86% (*P* < 0.01), the number of days taking antipyretics decreased by 80% (*P* < 0.01), children missed 81% fewer days of school (*P* < 0.01), and 77% fewer days of parental absence from work due to caregiving (*P* < 0.01) were observed
Marini et al (29).	2019	100	5–10	Children diagnosed with recurrent streptococcal tonsillitis and recommended by their doctor to receive adenotonsillectomy were recruited; 50 were given oropharyngeal probiotics for 90 days as the probiotic group, while 50 did not take any probiotics as the control group. They were followed up for 1 year for clinical observation since the start of oropharyngeal probiotic administration. All children received medication at the onset of tonsillitis during the study period according to standard clinical practice. Supplementation with oropharyngeal probiotics reduced the need for adenotonsillectomy in children by 72%. Despite the lower prevalence of adenotonsillectomy in the probiotic group during the study period, a lower incidence of pharyngeal tonsils, fewer days receiving antibiotics, and fewer days of school absence were observed in children in the probiotic group compared to the control group
Ilchenko et al (30).	2020	35	5–10	Thirty-five children diagnosed with recurrent streptococcal tonsillitis were recruited, 20 were adjuvantly treated with oropharyngeal probiotics for 30 days in addition to standard medication, and 15 were treated only with standard medication as the control group. Compared to the control group, administration of oropharyngeal probiotics significantly reduced the duration of tonsillitis-associated symptoms, including sore throat decreased by 36% (*p* < 0.05), fever decreased by 28% (*p* < 0.05), tonsil enlargement decreased by 43% (*p* < 0.05), throat ptosis decreased by 36% (*p* < 0.05), submaxillary lymph node enlargement decreased by 40% (*p* < 0.05), and palpated submaxillary lymph node pain decreased by 36% (*p* < 0.05). The detection rate of respiratory pathogens decreased with time over the 30-day oropharyngeal probiotic administration, including an 85% reduction in *Streptococcus pyogenes* (*p* < 0.05) and an 83% reduction in *Staphylococcus aureus* (*p* < 0.05). It is worth noting that more than half of the pathogens detected from the children with recurrent streptococcal tonsillitis recruited in this study carried antibiotic-resistant genes
Puhlik et al (31).	2021	90	15–40	Adolescents and adults diagnosed with *Streptococcus pyogenes*-associated recurrent pharyngotonsillitis were recruited, 30 were adjuvantly treated with oropharyngeal probiotics for 30 days in addition to standard medication, 30 were adjuvantly treated with oropharyngeal probiotics for 30 days in addition to standard medication and vacuum aspiration to remove nasal and tonsil secretions, and the other 30 only received standard medication as the control group. Follow-up was continued for up to 9 months. During the 9-month study period, oropharyngeal probiotics combined with tonsil vacuum aspiration had the best effect on preventing pharyngotonsil infections. The prevalence of pharyngotonsillitis in both groups receiving oropharyngeal probiotics was lower than that in the control group, while the pharyngeal detection rate for pathogenic streptococcal bacteria in swab was lower in patients adjuvantly treated with oropharyngeal probiotics compared to those who were not
Guo et al (32).	2022	97	3–10	Children with recurrent respiratory infections were recruited and randomly assigned to the probiotic group to adjuvantly treatment with oropharyngeal probiotics for 30 days, in addition to standard medication, or to the control group managed with standard treatment alone. Compared to children in the control group, adjuvant administration of oropharyngeal probiotics effectively protected children from new episodes of respiratory infections during both the 30-day intervention period and the 30-day follow-up period. Specifically, there was a 56% reduction in the prevalence of respiratory infections (*p* < 0.05), the duration of disease was shortened by 27% (*p* < 0.05), the average days of respiratory infection decreased by 68% (*p* < 0.05), the number of days taking antibiotics decreased by 97% (*p* < 0.05), and the number of days taking antiviral drugs decreased by 90% (*p* < 0.05). It is worth noting that, during the clinical observation period, no new respiratory infections were observed among children in the probiotic group from Day 10 of initiation to the end of the study (*p* = 0.002), while no antibiotic courses were prescribed among children in the probiotic group from Day 6 of initiation to the end of the study (*p* = 0.001). This observation suggests the protective effects of oropharyngeal probiotics for children with a history of recurrent respiratory infections

^a^ Only studies explicitly labeled the use of finished formulas rather than strains are selected by the panel.

^b^ Multicenter clinical study.

^c^ Retrospective study.

### 2.2 Otitis Media

Otitis media represents a significant burden on children, their families, and the healthcare system and is the major cause of hearing loss and serious life-long sequelae, such as behavior, attention (33), anxiety, learning, and speech-language problems in early and late childhood (34), if left untreated. Chronic and recurrent otitis media are recalcitrant to current therapies due to the formation of biofilms and intracellular biofilm pods by otopathogens on the middle ear mucosa and within the middle ear fluid (35). Antibiotics are generally used empirically for treating chronic suppurative otitis media, which may lead to the emergence of resistant bacterial strains (36). In addition, official recommendations differ regarding tympanostomy-tube placement that could favor the time to a first episode of acute otitis media and various episode-related clinical findings in young children with recurrent acute otitis media, but the rate of acute otitis media episodes, the percentage of episodes considered to be severe, and antimicrobial resistance among respiratory isolates were not significantly lower than with medical management (37). Relative abundances of potential pathogens, such as *Haemophilus influenzae*, *Streptococcus pneumoniae*, and *Moraxella catarrhalis* in the upper respiratory tract, might lead to further investigation into new preventive measurements for acute, secretory, and recurrent otitis media (38). The seven studies with evidence for the oropharyngeal probiotic formula, Bactoblis, providing a clinical benefit for otitis media management are listed in Table 2.

**Table 2:** Studies providing evidence that oropharyngeal probiotic Bactoblis can provide clinical benefit on otitis media management.

Reference	Year	Subjects	Age	Clinical evidence^a^
Di Pierro et al (22).	2012	82	3–12	Among 65 children diagnosed with recurrent streptococcal respiratory tract infections, 45 were assigned to take oropharyngeal probiotics for 90 days as the probiotic group, 20 did not take any probiotics as the recurrent control group, and another 17 children without recurrent respiratory tract infection were considered as the healthy control group. They were followed up for 6 months. The incidence of acute otitis media was reduced by 40% (*p* < 0.01) in children during oropharyngeal probiotic treatment compared with that in the previous year, while the incidence of acute otitis media was 2.16 times (*p* < 0.05) higher than that of the previous year in the recurrent control group and 1.95 times (*p* < 0.05) higher in the healthy control group. During the study period, it was 1.95 times higher than in the previous year (*p* < 0.05). During the 6-month follow-up period, the incidence of acute otitis media in children receiving oropharyngeal probiotics was 82% lower than that in the recurrent control group (*p* = 0.0278)
Di Pierro et al (39).	2015	22	3–9	Children diagnosed with recurrent otitis media with unilateral or bilateral middle ear effusion for more than 2 months were given oropharyngeal probiotics for 90 days. The incidence of acute otitis media episodes was reduced by 42.5% (*p* < 0.01) during probiotic administration compared with that in the previous year, while the left and right ear tone audiometry scores decreased by 55% and 66%, respectively (*p* < 0.01). Endoscopic scores for the left and right ear decreased by 39% and 40%, respectively (*p* < 0.05). Nasal endoscopy score decreased by 28.6% (*p* < 0.01). Tonsil test scores decreased by 36.8% (*p* < 0.01). In addition, the middle ear effusion was significantly improved in about 80% of children with recurrent otitis media after the 90-day oropharyngeal probiotic administration
Di Pierro et al (40).	2016	222	3	Healthy children who attended kindergarten for the first year were given oropharyngeal probiotics for 6 months. Compared with the control group who did not take any probiotics, the incidence of acute otitis media decreased by 45% (*p* < 0.01) during the 6 months of oropharyngeal probiotic administration and decreased by 67% (*p* < 0.05) during the 3-month follow-up period
Kryuchko et al (27).	2017^b^	66	3–10	No incidence of otitis media infection was observed among children with recurrent otitis media during the oropharyngeal probiotic administration for 30 days, while the incidence was 18% in children who did not take oropharyngeal probiotics during the same period
Di Pierro et al (41).	2018^b^	133	3–14	Compared with the previous year, the incidence of acute otitis media in healthy children without recurrent otitis media was reduced by 71% (*P* < 0.001) during the 1-year study period while oropharyngeal probiotics were administrated at intervals in the spring and autumn of the year
Havrylenko et al (42).	2019^b^	22	2–6	The incidence of acute otitis media decreased by 71% (*P* < 0.01) during the 30-day oropharyngeal probiotic administration in children diagnosed with secretory otitis media, compared to that of the previous year, while both the left and right otoscopy scores significantly decreased by 68% and 62%, respectively (*p* < 0.05). Nasal endoscopy score decreased by 59% (*p* < 0.01), and both the left and right middle ear effusion was significantly improved by 86% and 78%, respectively (*p* < 0.05)
Kryuchko et al (43).	2021	58	2–4	In the first year of kindergarten, healthy children who were given oropharyngeal probiotics had a reduced incidence of otitis media by 90% (*p* < 0.01) during the 90 days of probiotic administration compared to those who did not take any probiotics, while a reduced incidence of otitis media by 91% (*p* < 0.01) was observed during the followed-up period of 6 months compared with those who did not take probiotics

^a^ Only studies explicitly labeled the use of finished formulas rather than strains are selected by the panel.

^b^ Retrospective cohort study.

### 2.3 Acute respiratory tract infections

Respiratory viral infections are the most common type of acute respiratory infection, predisposing patients to secondary bacterial infections that often have a more severe clinical course. Antiviral immune responses induced by acute RTi are associated with dysbiosis in the respiratory tract, which in turn alters subsequent immune function against secondary bacterial infection or the dynamics of inter-microbial interactions, thereby enhancing the proliferation of potentially pathogenic bacterial species (44). Increased microbial diversity and growth rates of specific pathogens in the upper respiratory tract were observed. Oropharyngeal microbiota is a type specifically disrupted by the infection of influenza A virus (FluA), influenza B virus (FluB), respiratory syncytial virus (RSV), and human rhinovirus (HRV) (45), as well as omicron or other variant of SARS-CoV-2 viruses (46). Non-pathogenic commensal organisms colonized at nasopharynx and oropharynx possess the ability to interfere with the growth of potential pathogens such as *S. pneumoniae*, *H. influenzae*, and *M. catarrhalis*, the carriage of which increases during nasopharyngitis or pharyngitis, as well as symptomatic and asymptomatic viral URTi (47). Even though respiratory microbiota is shaped during the critical window of early life, season, and RTIs, evidence indicates a reduced niche differentiation preceding confirmed RTIs. This loss of ecological topography is further augmented by the start of daycare and linked to the consecutive development of symptomatic RTIs (48). In summary, restoring the loss of topography is linked to the prevention of subsequent development of RTi episodes. The four studies suggesting the clinical benefit of preventing acute RTi via administration of oropharyngeal probiotic Bactoblis are listed in Table 3.

**Table 3:** Studies providing evidence of the clinical benefit of preventing acute RTi via administration of oropharyngeal probiotic Bactoblis.

Reference	Year	Subjects	Age	Clinical evidence^a^
Pierro et al (40).	2016	222	3	Healthy children who entered kindergarten in the first year were assigned to take oropharyngeal probiotics for 6 months, while the others who did not take any probiotics as the control group were followed up for 3 months. The incidence of pharyngeal tonsillitis decreased by 67% (*p* < 0.01) during the 6 months of oropharyngeal probiotic administration compared to the control group. The incidence of pharyngeal tonsillitis showed a decreasing trend during the follow-up period
Pierro et al (41).	2018^b^	133	3–14	Healthy children were given oropharyngeal probiotics for 3 months in the spring and autumn of the year and followed up for a year. The prevalence of streptococcal pharyngotonsil infection in children during the 1-year study period decreased by 89% compared with that of the previous year (*p* < 0.001), the prevalence of non-streptococcal pharyngotonsil infection decreased by 94% (*p* < 0.001), the number of days of antibiotic use decreased by 88% (*p* < 0.01), the number of days of antipyretic drug use decreased by 85% (*p* < 0.01), the number of days of absence from school due to illness decreased by 85% (*p* < 0.01), and the number of days of parental absence from work due to caregiving decreased by 75% (*p* < 0.01) compared with those of the previous year
Kryuchko et al (43).	2021	58	2–4	In the first year of kindergarten, healthy children were given oropharyngeal probiotics for 90 days and were followed up for 6 months. Compared to those who did not take any probiotics as the control group, the prevalence of pharyngeal tonsillitis decreased by 92% (*p* < 0.01), bronchitis decreased by 63% (*p* < 0.01), rhinitis decreased by 61% (*p* < 0.01), and laryngitis decreased by 79% (*p* < 0.01) in children during oropharyngeal probiotic administration. During the 6-month follow-up period, the prevalence of pharyngeal tonsillitis decreased by 82% (*p* < 0.01), bronchitis decreased by 65% (*p* < 0.01), rhinitis decreased by 63% (*p* < 0.01), and laryngitis decreased by 71% (*p* < 0.01) among children in the probiotic group compared to those in the control group. Throughout the study period, children in the probiotic group were significantly less likely to be prescribed medications and absent from school than those in the control group, of which 65% (*p* < 0.01) fewer days of antibiotic use, 73% (*p* < 0.01) fewer days of antipyretic drug use, and 61% (*p* < 0.01) fewer days of absence from school were observed
Kryuchko et al (49).	2021	62	0.5–2	Healthy infants and toddlers were randomly assigned into the probiotic group to take oropharyngeal probiotics for 30 days and the control group to not take any probiotics as the control group and were followed up for 3 months. Healthy infants and toddlers who took oropharyngeal probiotics were better protected from respiratory infections throughout the study period compared to controls, with a 65% (*p* < 0.01) reduction in the prevalence of viral acute respiratory infections, a 76% (*p* < 0.01) reduction in the prevalence of secondary bacterial respiratory infections, an 86% (*p* < 0.01) reduction in the number of otorhinolaryngologic outpatient visits, an 81% (*p* < 0.01) reduction in days of antibiotic use, and a 55% (*p* < 0.01) reduction in days of antipyretic drug use

^a^ Only studies explicitly labeled the use of finished formulas rather than strains are selected by the panel.

^b^ Retrospective cohort study.

### 2.4 Chronic adenoiditis and chronic tonsillitis

Repeated use of antibiotics is common during the treatment of tonsillitis, and prior antibiotic use is a major contributor to subsequent antibiotic prescribing (50), except for recurrent antibiotic prescriptions. Tonsillectomy remains a common pediatric surgery for recurrent and chronic tonsillitis or recurrent otitis media; however, severe postoperative pain is common, and some patients will have postoperative complications of bleeding (51). Importantly, a cohort study of 1.2 million patients followed up for 30 years showed that childhood adenoidectomy or tonsillectomy was associated with a significantly increased relative risk of respiratory infections and allergies later in life. Increases in long-term absolute disease risks were considerably larger than changes in risk for the disorders these surgeries aim to treat, suggesting that it is important to consider long-term risks when making decisions to perform tonsillectomy or adenoidectomy (52). Children who had adenoidectomy for adenoid-related diseases are mostly due to obstructive sleep-disordered breathing, otitis media with effusion, and chronic sinusitis, while respiratory pathogens *H. influenzae*, *S. aureus*, *S. pneumoniae*, and *M. catarrhalis* are commonly found in adenoids (53). Surgical removal of adenoids and tonsils to treat obstructed breathing or recurrent middle ear infections remains a common pediatric procedure; however, a meta-analysis of current literature that included more than a thousand subjects demonstrated that pediatric sleep apnea is often not cured by tonsillectomy and adenoidectomy (54).

Chronic adenoiditis occurs frequently in children and is complicated by the subsequent development of recurrent or chronic middle ear diseases, such as recurrent acute otitis media, persistent otitis media with effusion, and chronic otitis media that fail to respond to traditional antibiotic therapy, which may predispose a child to long-term functional sequalae and auditory impairment (55).

In the treatment of children with chronic adenoiditis, it is necessary to take into account the features of the normal microbiota of the nasopharynx, by acting on opportunistic and pathogenic microorganisms. Favorable conditions for stimulating the growth and development of representatives of the indigenic microbiota can be created, which in turn will contribute to the patient's speedy recovery from chronic adenoiditis and absence of relapses (56). Further investigation of individual microbiomes in a longitudinal design with implantation of protective oropharyngeal probiotics may have the potential to lead to new strategies as an alternative to adenoidectomy. The four studies demonstrating that the oropharyngeal probiotic Bactoblis can provide clinical benefit on chronic adenoiditis and tonsillitis management are listed in Table 4.

**Table 4:** Studies providing evidence that oropharyngeal probiotic Bactoblis can provide clinical benefit on chronic adenoiditis and tonsillitis management.

Reference	Year	Subjects	Age	Clinical evidence^a^
Karpova et al (57).	2015	219	6–7	The incidence of acute adenoiditis decreased by 44% in children diagnosed with chronic adenoiditis during the 30-day oropharyngeal probiotic administration along with nasal irrigation, compared to those only treated with nasal irrigation as conservative treatment, while it decreased by 74% during the 90-day follow-up period. The incidence of acute otitis media, a common complication of chronic adenoiditis in children receiving oropharyngeal probiotics, also decreased by 62%
Marushko et al (58).	2018^b^	54	9–14	Children with chronic tonsillitis manifested symptoms more than twice a year and were treated with conservative nasal irrigation, of whom 70% had symptoms of enlarged tonsils, who were treated with oropharyngeal probiotics for 30 days in May and 30 days in September. During the observation period of 30 and 180 consecutive days from the beginning of probiotic administration, various clinical symptoms related to chronic tonsillitis improved over time, including a 14% and 52% reduction in the prevalence of enlarged tonsils at 30 and 180 days, respectively (*p* < 0.05); the prevalence of general discomfort decreased by 57% and 74%, respectively (*p* < 0.05); low-grade fever decreased by 88% and 80%, respectively (*p* < 0.05); arthralgias decreased by 65% and 55%, respectively (*p* < 0.05); palpated lymph node enlargement decreased by 23% and 47%, respectively (*p* < 0.05); leucocytosis decreased by 89% and 87%, respectively (*p* < 0.05); leukemia-like reactions decreased by 43% and 91%, respectively (*p* < 0.05); C-reactive protein both increased by 97% (*p* < 0.05); and allergic manifestations caused by aggravation of tonsillitis decreased by 82% and 100%, respectively (*p* < 0.05). During the study period, the detection rate of pathogenic bacteria on the pharyngeal tonsillar mucosa also decreased with time, including a reduction of beta-hemolytic Group A *Streptococcus* (BHSGA) by 82% and 67% at 30 and 180 days, respectively (*p* < 0.05), and BHSGA associated with *Staphylococcus aureus* decreased by 100% and 71% at 30 and 180 days, respectively (*p* < 0.05)
Gavrilenko et al (59).	2018	57	6–10	Children diagnosed with recurrent pharyngeal tonsillitis were divided into three subgroups, including ones of chronic tonsillitis, hemolytic streptococcal infection, and swollen pharyngeal tonsillitis. Children in the three subgroups were further randomly assigned to the probiotic group to take oropharyngeal probiotics for 30 days and the control group to take no probiotics. Children who received oropharyngeal probiotics for 30 days had an 85% (*p* < 0.05) reduction in the incidence of hemolytic streptococcal respiratory infections throughout the clinical observation period of 5 months compared to the same period in the previous year, while there was no significant difference in the control group. Chronic tonsillitis-related symptoms, such as shortness of breath, mouth breathing during sleep, snoring, cough (mainly during the night and in the morning), nasal congestion, and hearing loss, showed significant improvement since Day 10 after the start of the study. There was a decreased trend of detection rate for *Staphylococcus aureus*, *Streptococcus pneumoniae*, *Streptococcus pyogenes*, *Streptococcus pneumococcus*, and other pathogenic bacteria in the throat samples among children in the chronic tonsillitis subgroup during the 30-day oropharyngeal probiotic administration compared with those in the control group
Marini et al (29).	2019	100	5–10	Children diagnosed with chronic streptococcal tonsillitis who were recommended to receive adenotonsillectomy were randomly assigned to take oropharyngeal probiotics for 3 months while the others did not take probiotics as the control group and were followed up for another three quarters. In addition, 72% less of children taking oropharyngeal probiotics underwent adenotonsillectomy throughout the study period compared to those in the control group. Furthermore, compared to those in the control group, a reduction of 64%, 50%, 39%, and 43% (*p* < 0.01), respectively, in the incidence of tonsillitis during each quarter of the study period was observed in children taking oropharyngeal probiotics. The number of days receiving antibiotics also decreased by 81%, 60%, 58%, and 53% (*p* < 0.01) during each quarter, respectively, in children taking oropharyngeal probiotics compared with the control group, while the days of absence from school decreased by 54% in the probiotic group compared with the control group during the study period

^a^ Only studies explicitly labeled the use of finished formulas rather than strains are selected by the panel.

^b^ Retrospective cohort study.

### 2.5 PFAPA (periodic fever, aphthous stomatitis, pharyngitis, and adenitis) syndrome

The microbiota of the tonsils removed from PFAPA patients differed significantly from those of the non-PFAPA patients, indicating that tonsillar microbiota may play a role in triggering the inflammatory processes that lead to symptoms of PFAPA (60). Furthermore, tonsil dysbiosis may be associated with altered antimicrobial peptide expression on tonsil surface epithelium as in other autoinflammatory diseases which was not evident in recurrent tonsillitis (61). The two studies evidencing that oropharyngeal probiotic Bactoblis can provide clinical benefit on PFAPA management are listed in Table 5.

**Table 5:** Studies evidencing that oropharyngeal probiotic Bactoblis can provide clinical benefit on PFAPA management.

Reference	Year	Subjects	Age	Clinical evidence^a^
Francesco et al (62).	2016^b^	4	5–10	The first study showing clinical evidence that significant improvement in PFAPA clinical symptoms was observed during both the 90-day Bactoblis administration and 30-day follow-up period, including fever, oral ulcers, the frequency and days of pharyngitis, and adenoid enlargement, resulting in not only a decrease in the use of medications, such as cortisone and Ibuprofen, but also a significantly improved life and sleep quality during the 4-month study period
Torreet al (63).	2023^c^	85	1–8	Eighty-five children with PFAPA at a mean age of 2 years who had taken Bactoblis at a mean age of 4.58 years were selected from the International Autoinflammatory Diseases Alliance Registry (AIDA). The mean duration of Bactoblis administration was 6 months, and the median age of PFAPA illness before starting Bactoblis administration was 19 months. During the period of taking oropharyngeal probiotics, children with PFAPA experienced significantly reduced episodes of febrile (85% of children had reduced febrile episodes from a median of 13 episodes to 5 episodes; *p* < 0.001), significantly reduced days of febrile (77.6% of the children experienced reduced days of febrile reduced from a median of 4 days to 2 days; *p* < 0.001), while their body temperature during febrile significantly decreased by an average of 1° (40℃ vs. 39℃; *p* < 0.001), the incidence of symptoms during PFAPA episodes was significantly improved, including headache (*p* < 0.05), pharyngitis (*p* < 0.001), oral ulcer (*p* < 0.001), adenoid enlargement (*p* < 0.001), abdominal pain (*p* < 0.001), and joint and muscle pain (*p* < 0.001), resulting to a significant reduction in the needs for drugs, such as a 60% reduction in the use of the anti-inflammatory betamethasone or other equivalent steroid drugs (*p* < 0.001). No adverse reactions were reported throughout the study period

^a^ Only studies explicitly labeled the use of finished formulas rather than strains are selected by the panel.

^b^ Prospective study.

^c^ retrospective cohort study.

## 3 Recommendations for future research on oropharyngeal probiotics

### 3.1 Allergic rhinitis

Repeated cycles of infection-associated lower airway inflammation drive the pathogenesis of persistent wheezing disease in children. The occurrence of viral acute is accompanied by a shift in the nasopharyngeal microflora toward dominance by a small range of pathogenic bacterial genera. However, this change frequently precedes the detection of viral pathogens and acute symptoms. Colonization of illness-associated bacteria coupled with early allergic sensitization is associated with persistent wheeze in school-aged children, which is the hallmark of the asthma phenotype (64). Furthermore, pediatric chronic rhinosinusitis is a condition commonly encountered in otolaryngology practice, a proportion of which progresses from acute bacterial sinusitis induced by upper respiratory tract infections (65). Intranasal corticosteroids remain the first-line treatment for chronic rhinosinusitis. The study results of the effects of intranasal corticosteroids on the composition of the respiratory microbiome were highly heterogeneous (66), due to their immunosuppressive properties. It is worthwhile considering the possibility of the long-term use of inhaled corticosteroids associated with an increased risk of bacterial infections (67). Children admitted with asthma exacerbations harbor a microbiome characterized by overgrowth of *Staphylococcus* and oral microbes and an underrepresentation of beneficial niche-appropriate commensals (68, 69), which also present seasonally (70). Restoring the beneficial nasopharyngeal microbiota could be an alternative approach for self-management in pediatric patients with allergic respiratory conditions. Evidence from observational studies of children attending daycare revealed that nasopharyngeal probiotic Bactoblis administration in children was associated with an increased abundance of commensal *S. salivarius* in saliva and a lower abundance of otopathogens, *Moraxella*, in the nasopharynx which is strongly associated with the exacerbation of asthma (71). More research on oropharyngeal microflora intervention might help improve the quality of life for people with allergic rhinitis and reduce the risk of developing respiratory infections during high allergy seasons.

### 3.2 Systemic autoimmune diseases

Despite decades of research, systemic autoimmune diseases (SADs) continue to be a major global health concern, and the etiology of these diseases remain unclear. Oral microbial dysbiosis has been identified in SADs including systemic lupus erythematosus (SLE), rheumatoid arthritis (RA), and Sjögren's syndrome (SS), although the dysbiosis features were different among studies (72). Although several investigations failed to establish causal relationships, with the exception of Group A *Streptococcus* in rheumatic arthritis, microbial contributions to SAD initiation and propagation are plausible and likely, especially via the connective tissue and primary vasculitides (73). Indeed, accumulating evidence shows that antimicrobial peptides are induced or upregulated by dysbiosis in SADs while the host immune system attacks self-tissues with complex pathogenesis (74). Juvenile idiopathic arthritis (JIA) is the most common chronic rheumatic disease in children. Infectious agents are suspected to be environmental triggers involved with molecular mimicry between bacterial molecules and self-antigens (75). In addition, DNA from oral and gut microbiota can be identified in RA synovium, possibly due to the translocation through circulation from the oral cavity (76, 77).

### 3.3 Occupational respiratory health for health care workers

The World Health Organization (WHO) and the International Labour Organization (ILO) have published a new guide on developing and implementing stronger occupational health and safety programs for health workers in 2022. Even before the COVID-19 pandemic, the health sector was among the most hazardous sectors to work in due to the suffering from infections and allergies from the working environment (78). Since individuals spend most of their lives at work, occupational exposures may have an impact on the microbiota. Chronic respiratory diseases are ranked as the most common occupational disease (79), and the concept of WORKbiota has been recently emphasized by European scientists in 2022 (80). A randomized controlled study conducted during the early COVID-19 pandemic showed that oropharyngeal probiotic Bactoblis protects frontline healthcare workers from respiratory tract infections during the month of taking care of critically ill COVID-19 patients (81). Establishing a homeostasis status of respiratory microflora has been recognized as a safe and positive approach to achieving occupational-related respiratory health by international experts (82). Occupational allergens are one of the risk factors for allergic rhinitis (83), and healthcare workers are exposed to a range of allergens including cleaners and disinfectants, natural rubber latex, and various medications. Studies have shown that exposed healthcare workers are at risk for work-related rhinitis and asthma. For example, high prevalence rates of occupational asthma are found in nurses (10.7%) in Japan according to the Japanese Guidelines for Occupational Allergic Diseases published in 2020 (84). A review of cross-sectional studies indicated that occupational rhinitis affects 10%∼60% of healthcare workers, and occupational anaphylaxis was most frequently triggered by natural rubber latex, chemicals, disinfectants, and medications (85). The inflammatory response of allergic rhinitis continues to interact with the imbalance of nasal flora. Exposure to allergens will induce changes in the bacterial flora of the nasal mucosa, leading to acute sinusitis, nasal eosinophilia, and more serious nasal symptoms, which will reduce the quality of life (70). In addition to occupation-related allergens, the adhesion or colonization of specific opportunistic pathogens in the nasal mucosa is also an important risk factor for inducing chronic airway inflammation leading to allergic respiratory diseases (86). It has been reported that the oropharyngeal colonization of *Streptococcus pneumoniae* and *Haemophilus influenzae* in healthcare workers working in hospitals is as high as two times compared to non-healthcare workers (87, 88). It has also been documented that healthcare workers (89), medical laboratory staff due to direct and dense contact with the pathogens, and those living with hospital staff have a higher prevalence of *Methicillin*-resistant *S. aureus* (MRSA) nasal colonization. The isolates also appeared more virulent while all isolates were *β*-lactamases positive (90). Another study indicated that the carriage rates of *S. aureus* and MRSA among surgical HCWs (32.4%) and nurses (30.8%) were relatively higher, while the highest MRSA rate was detected in nurses (91). The presence of *Staphylococcus* spp. was more prevalently in the hands of HCWs working in the internal medicine ward and the surgical ward, which is about six times compared to personnel in the neonatal unit, while those with multidrug-resistant or extensively drug-resistant strains were isolated (92). It was also demonstrated that *Staphylococcus* spp. were most frequently (40%) isolated from the cellphones of hospital staff, while Gram-positive isolates were all susceptible to the antibiotic used and Gram-negative isolates were all resistant to ceftazidime (93). The establishment of a balanced and healthier respiratory microflora through the intervention of oropharyngeal probiotics among healthcare workers and their families is expected to help protect their long-term respiratory health and reduce the risk of transmission of resistance genes to their family members.

### 3.4 Mode of action of oropharyngeal probiotics on protecting hosts from respiratory infections

Antiviral microbiome mechanisms include the following: (1) enhancement of mucosal barrier function which provides a physical barrier between invasive pathogens and host epithelial cells where the tight junctions and mucosal permeability are maintained by commensal microbiome; (2) antimicrobial compounds, such as bacteriocins, produced by commensal microbiome; (3) inhibition of viral attachment to host epithelial cells by various cross-immune reactions; and (4) modulation of the immune system, such as downregulating inflammatory immune pathways and/or enhancing innate and/or adopted immune pathways and cytokine signaling (94). In children with regurgitation and microaspiration syndromes, the use of oropharyngeal probiotic Bactoblis had a pronounced positive effect on the respiratory microbial composition, of which the pathogenic *Staphylococcus aureus*, *Staphylococcus epidermidis*, *Streptococcus pyogenes*, and *Klebsiella pneumoniae* were significantly reduced (95). A study conducted on young athletes performing a high-intensity training program showed that short-term administration of oropharyngeal probiotic Bactoblis significantly increased salivary immunoglobulin (sIgA) secretion in young athletes, an indication of a potential immune enhancement (96). Since the oropharynx is a primary source of the lung microbiota community which contributes to the susceptibility to viral infections, a preliminary study showed that a 14-day administration of oropharyngeal probiotic Bactoblis reduced the mortality rate of hospitalized COVID-19 patients, improving the clinical parameters associated with pulmonary inflammation caused by viral infections and immune responses (97). These clinical evidence-based findings reflected these mechanisms and suggested that improving the respiratory microbiota may enhance immune function and anti-inflammatory effects. Therefore, more research on respiratory microecological intervention and immune regulation mechanisms will provide a more solid theoretical foundation for clinical application and evidence-based medicine.

### 3.5 Reducing the carriage of antibiotic resistance genes in human

The rise of antibiotic resistance and a dwindling antimicrobial pipeline have been recognized as emerging threats to public health (98). Enormous therapeutic challenges may present in specific groups of children who have a higher risk of acquiring antibiotic-resistant genes. For example, a major proportion of pneumococci isolated from the nasopharyngeal aspirates of inpatient children with respiratory infections were resistant to more than three types of antibiotics in China (99). Resistant bacteria are present in significant amounts in the adenoids of children with middle ear disease and rhinosinusitis symptoms compared with healthy children (100). A large-scale multicenter study showed that the resistance rate of ampicillin and azithromycin in *H. influenzae* isolated from the respiratory tract in Chinese children showed an increasing trend through the years, and the major multidrug resistance pattern was resistant to *β*-lactams, macrolides, and sulfonamides (101). Group A *Streptococcus* (GAS) is an important cause of acute pharyngitis, and its positive rate in throat culture was about 20% in younger children (102). A retrospective study conducted for 20 years in Taiwan indicated that there was a significant increase by three times to reach about 60% (*p* < 0.0001) in macrolide-resistant Group A *Streptococcus* in children with URTi, especially for those diagnosed with scarlet fever (103). The outcome was supported by a similar result from a study conducted in Beijing that prevalent strain of Group A *Streptococcus* collected from pediatric outpatients who were diagnosed with scarlet fever has a high resistance rate to macrolides at over 90% (104). In a recent study, a strong seasonal epidemiological association between respiratory syncytial virus (RSV) and *Streptococcus pneumoniae* (pneumococcus) was confirmed by a parallel decrease and a subsequent resurgence during and after the COVID-19 pandemic, respectively (105). Considering the increased concurrent pediatric RSV infections and GAS pharyngitis and the high prevalence of resistant genes in children, the intervention of oropharyngeal probiotics has great potential to support the management of an important public health condition. From 1990 to 2021, deaths due to antimicrobial resistance increased by over 80% among individuals aged 70 years and older globally (106). This indicates the growing challenge of antibiotic resistance, which is now recognized as a global public health crisis. It is essential that everyone, from individuals to countries, actively respond to and take action to address this emergency. Clinicians should play a central role in addressing antibiotic resistance, and medical training is a key factor in this effort. However, there remain gaps between the knowledge and practices among healthcare workers regarding antibiotic use. There is a clear need for multifaceted interventions targeting the public to improve behaviors of rational use of antibiotics.

## 4 Conclusion

The panel conducted a technical review of evidence-based clinical studies and completed the first expert consensus on the adjuvant use of oropharyngeal probiotics for the management of pediatric respiratory tract infections and otitis media. This consensus was based on the clinical studies that explicitly labeled the use of evidence-based finished formulas rather than formulas with only strains described of which the clinical benefit is undefined.

The consensus process aims to help doctors better understand how to use the evidence-based oropharyngeal probiotic Bactoblis as a dietary supplement and an assistive tool, adjunctively with standard treatment, to manage pediatric respiratory health. This is important for managing refractory pediatric recurrent and chronic respiratory tract infections, such as recurrent and suppurative otitis media, recurrent tonsillitis, chronic adenoiditis, etc. When adjuvantly or prophylactically supplemented, oropharyngeal probiotics can safely reduce the incidence of respiratory tract infections and shorten the course of infectious episodes. Meanwhile, children and parents or caregivers can benefit from reduced absence from school due to illness, absence due to caregiving, and reduced need for prescriptions of antibiotics and antiviral drugs. This expert consensus can be considered as a widely applicable strategy for self-health management that is accepted according to the patient's active will. The recommendation of oropharyngeal probiotics is not intended to replace any standard treatment.

## 5 Annotation

The oropharyngeal probiotic Bactoblis, which is available in China, contains *Streptococcus salivarius* subsp. *thermophilus* Biohalo23. The recommended daily effective dosage is no less than 1 billion CFUs for individuals of all ages, including toddlers, children, adolescents, and adults.
